# A protocol for assessing smallholder farmers’ climate resilience: A guidance for researchers to construct a robust multidimensional domain

**DOI:** 10.1016/j.mex.2025.103763

**Published:** 2025-12-13

**Authors:** Imade Yoga Prasada, Jangkung Handoyo Mulyo, Hani Perwitasari, Agus Dwi Nugroho

**Affiliations:** a Doctoral School of Agricultural Sciences, Faculty of Agriculture, Universitas Gadjah Mada, Yogyakarta, Indonesia; b Study Program of Agribusiness, Faculty of Science and Technology, Universitas Putra Bangsa, Kebumen, Indonesia; c Study Program of Agricultural Economics and Agribusiness, Department of Agricultural Socio-economics, Faculty of Agriculture, Universitas Gadjah Mada, Yogyakarta, Indonesia

**Keywords:** Climate resilience, Multidimensional domain, Smallholder farmers, Assessment

## Abstract

In recent years, the theoretical frameworks on climate resilience of smallholder farmers have continued to develop. This has encouraged the emergence of a variety of domains, indicators, and items. Meanwhile, inappropriate construct domains, indicators, and items can lead to biased research findings. Therefore, developing a robust construct domain, indicators, and items requires a clear, easily understood, and statistically accountable protocol. This protocol emphasizes the formation of a framework for conducting a statistical approach to assess smallholder farmers’ climate resilience.

## Graphical abstract

**Figure:**
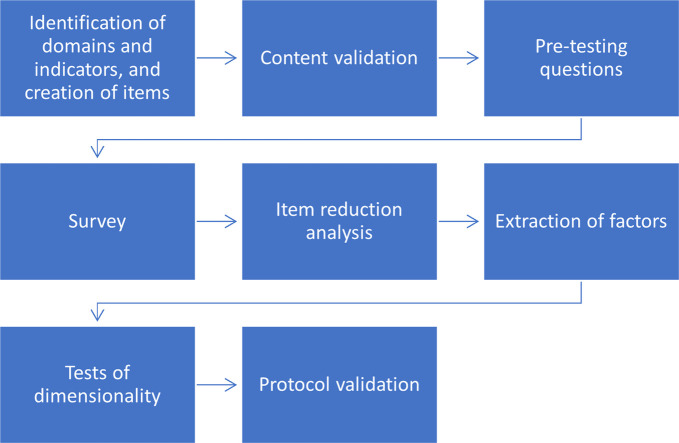

**Specifications table**

**Table:** 

**Subject area**	Environmental Science
**More specific subject area**	*Climate resilience assessment of smallholder farmers*
**Name of your protocol**	*Self-assessment protocol for smallholder farmers’ climate resilience*
**Reagents/tools**	*Observation, interview templates, Excel program, Stata program, SmartPLS program*
**Experimental design**	*Not applicable*
**Trial registration**	*Not applicable*
**Ethics**	*This study followed ethical standards for research involving human subjects. Informed consent was obtained from all respondents, and participation was voluntary and anonymous.*
**Value of the Protocol**	1. The protocol provides a clear framework for building a robust multidimensional domain for assessing smallholder farmers’ climate resilience.
	2. The protocol is easily adaptable to various types of qualitative and quantitative research.
	3. The protocol allows researchers to provide valid and reliable statistical justification when using a posteriori-defined domains.
	4. The protocol can be run using simple tools and statistical software that are already widely known to researchers.

## Background

The definition of resilience continues to evolve over time. Folke et al. [1] state that resilience can be divided into two types: general resilience and specific resilience. General resilience refers to the ability to face all kinds of challenges, including entirely new ones, whereas specific resilience refers to the ability to overcome specific challenges, such as climate change.

Resilience to climate change, or climate resilience, can be interpreted as a person's ability to recover, reorganize, and thrive in the face of external shocks like natural disasters or extreme weather conditions[2,3]. There have been numerous advancements in the theories and methods for assessing climate resilience. Holling [4] introduced the theory of resilience in ecological systems, where the concept explains how ecological systems can withstand a variety of shocks, reorganize while maintaining their function and structure, and develop more complex systems. Therefore, the level of resilience based on this theory can be determined using multidimensional domains, including the domains of absorptive capacity, adaptive capacity, and transformative capacity.

Furthermore, Berkes & Folke [5] developed a social-ecological systems theory that shows the relationship between ecological systems and social systems, allowing the level of resilience to be determined by the interaction between both systems. According to this theory, resilience is measured by considering several domains, including diversity and redundancy, connectivity, learning and adaptation, and social participation. Then, Majale [6] emphasized that the resilience of farmer households can be formed if the sustainable livelihoods framework is appropriately implemented. This framework consists of several domains, including the availability of human resources, social and physical conditions, financial capabilities, and natural resources. The use of domains in measuring the smallholder farmers’ climate resilience is further developed and determined a posteriori (based on empirical facts in each research area).

Unfortunately, a posteriori domains have several weaknesses: insufficient predictive ability[7], allow for retrospective bias that can lead to incorrect conclusions[8,9], and limited temporal validity[10,11]. However, domains determined a posteriori have the advantage of being relevant[12,13] and adaptable to the area or issue being studied[14]. Therefore, a clear protocol is required to develop a robust multidimensional domain.

## Description of protocol

**Fig. 1:**
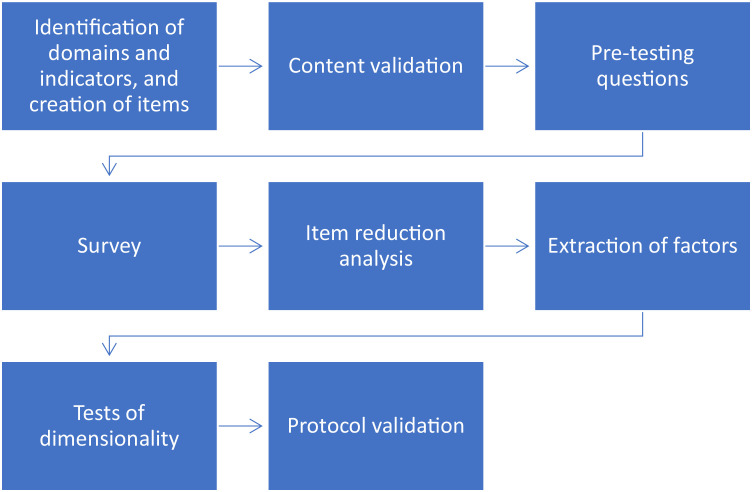
A protocol for smallholder farmers’ climate resilience assessment.

There are various approaches to assessing the climate resilience of smallholder farmers. However, most of these approaches utilize latent constructs, which necessitate the compilation of appropriate domains and items, as well as statistical testing to avoid bias in research results[15,16]. Therefore, the self-assessment protocol for climate resilience of smallholder farmers can be a solution to these issues. This protocol produces a robust multidimensional domain by combining several statistical methods into a single. Furthermore, the protocol that is compiled has two benefits: First, can be contributed to the provision of a framework for conducting multidimensional domains assessments, especially smallholder farmers’ climate resilience assessment (Fig. 1); second, can be used in various types of self-assessments involving multidimensional domains because it is compiled follows scientific principles (can be tested statistically)[17].

Protocol implementation is relatively cheap since it only requires purchasing a license for the necessary data analysis software (apart from the cost of field data collection, which will depend on sample size and geographic variation). Our protocol is inclusive since it may be applied to both genders. Furthermore, the protocol can be replicated across different geographic settings, allowing for flexible use of domains, indicators, and items based on the geographic location and unique characteristics of each smallholder farmer. Protocols can consist of several specific methods where the method used must be easy and cheap, but at the same time capable of providing the best outcomes[18].

### Identification of domains and indicators, and creation of items

**Fig. 2:**
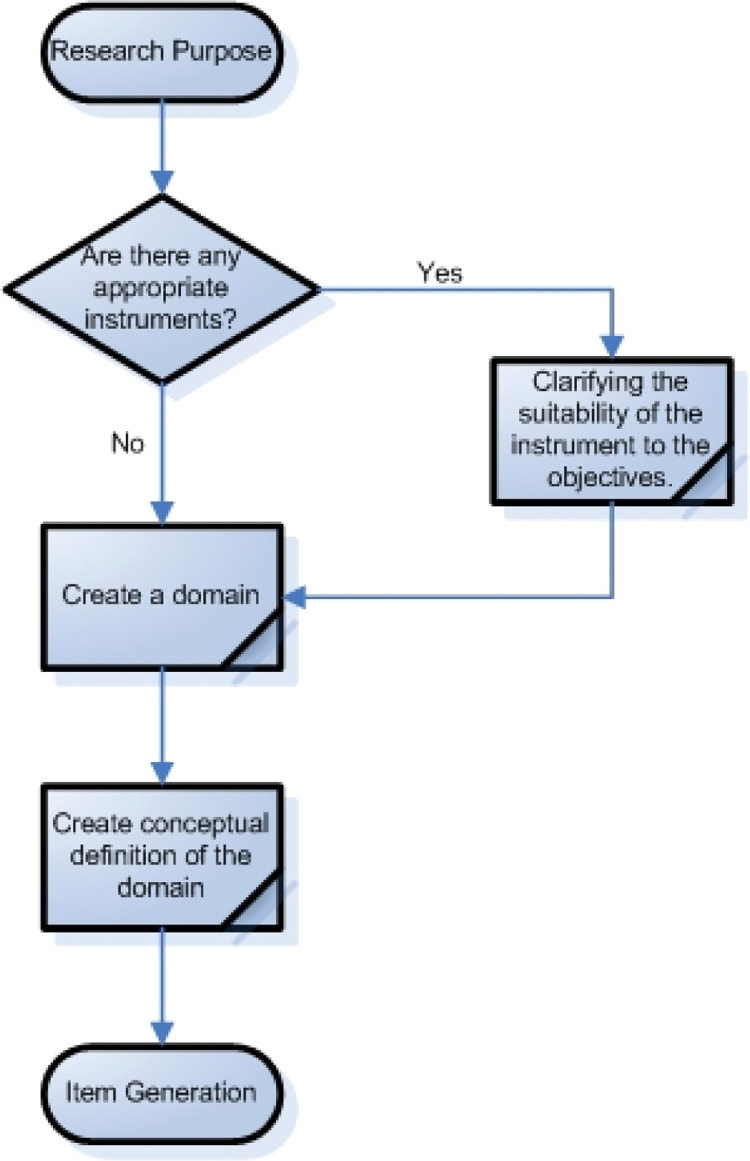
Steps to construct domains and items.

The first stage in this protocol is defining the domain to be measured. The construct of a domain refers to the theory, concept, attribute, or unobserved behavior that is the subject of the study[19]. Therefore, the domain can be determined a priori or a posteriori. Determination of the domain a priori is based on the theoretical framework compiled by the researcher[20], while the domain determined as a posteriori is a domain formed based on empirical facts from each study location[21]. Next, several processes are taken to determine the domain. First, determine the research objectives (Fig. 2). Second, find an instrument that aligns with these objectives. If an existing instrument meets the research objectives, the study must explain how well it fits the field conditions. Third, the researcher constructs multidimensional domains simultaneously. In this step, the researcher can also formulate appropriate indicators for each domain. Fourth, each domain is given a conceptual definition. Fifth, the researcher determines the correct items to measure each domain.

It should be noted that item construction is essential to ensure the robustness of the multidimensional domain. Therefore, item construction can be determined using three approaches, namely the deductive approach, the inductive approach, or a combination of both[22]. The deductive approach focuses on describing the domain or indicator that has been constructed, while the inductive approach emphasizes the response of all members of the constructed domain. The combined approach of the deductive and inductive approaches focuses on the conceptual definition of each domain, while also considering the responses of everyone based on the observation results. Furthermore, each item needs to be assessed using a specific measurement scale, either using a Likert scale or using other scoring techniques[23].

In assessing smallholder farmers’ climate resilience, a theoretical framework related to climate resilience and sustainable development can be used to gather the appropriate domains, indicators, and items. Dixon et al. [24] explained that resilience has three basic principles, namely perceived robustness, adaptability, and transformability capacities. In addition, based on the theory of sustainable development, three primary aspects—economic, social, and environmental—can be combined to generate resilience[25]. These three aspects are expected to create positive interactions and support each other. Therefore, these three aspects serve as domains to determine the level of smallholder farmers’ climate resilience (Table 1.).

**Table 1:** Domains, indicators, items, and measurement scales for smallholder farmers' climate resilience assessment.

**Domain**	**Definition**	**Indicator**	**Item**	**Scale**
Economic (EC)	The economic capacity of farming households to anticipate, respond to, and recover from the impacts of climate change.	Production	EC1: Despite extreme weather, the farm under my management can still produce well.	Likert scale, where 1 corresponded to “I strongly disagree” and 5 to “I strongly agree”
			EC2: Despite extreme weather, food remains available for all family members.	Likert scale, where 1 corresponded to “I strongly disagree” and 5 to “I strongly agree”
			EC3: Despite extreme weather, I find it very easy to market the yield.	Likert scale, where 1 corresponded to “I strongly disagree” and 5 to “I strongly agree”
		Income	EC4: Despite extreme weather, I get a decent price for my harvest sales transactions.	Likert scale, where 1 corresponded to “I strongly disagree” and 5 to “I strongly agree”
			EC5: Despite extreme weather, my off-farm income is unaffected.	Likert scale, where 1 corresponded to “I strongly disagree” and 5 to “I strongly agree”
Environment (EN)	The physical, biological, and ecological aspects that shape the agricultural systems managed by farmers.	Pest management	EN1: I always use pesticides according to the instructions for use.	Likert scale, where 1 corresponded to “I strongly disagree” and 5 to “I strongly agree”
		Water quality	EN2: I use odorless, tasteless, and colorless water for my irrigation operations.	Likert scale, where 1 corresponded to “I strongly disagree” and 5 to “I strongly agree”
		Soil quality	EN3: My land has good soil fertility for cultivation, which reduces fertilizer usage.	Likert scale, where 1 corresponded to “I strongly disagree” and 5 to “I strongly agree”
			EN4: Excessive use of chemical fertilizers on the agricultural land under my management will decline soil quality in the long term.	Likert scale, where 1 corresponded to “I strongly disagree” and 5 to “I strongly agree”
		Agricultural system	EN5: I prefer conventional farming systems compared to sustainable farming systems.	Likert scale, where 1 corresponded to “I strongly disagree” and 5 to “I strongly agree”
			EN6: I implement intercropping farming systems to maintain soil quality.	Likert scale, where 1 corresponded to “I strongly disagree” and 5 to “I strongly agree”
Social (SO)	Social aspects that influence the ability of farming households to anticipate, respond to, adapt to, and recover from the impacts of climate change.	Access to information	SO1: It is very easy for me to access agricultural information, especially regarding the weather.	Likert scale, where 1 corresponded to “I strongly disagree” and 5 to “I strongly agree”
			SO2: I get reliable agricultural information.	Likert scale, where 1 corresponded to “I strongly disagree” and 5 to “I strongly agree”
		Farmer institutions	SO3: In my opinion, farmer groups play an essential role in increasing climate change adaptation.	Likert scale, where 1 corresponded to “I strongly disagree” and 5 to “I strongly agree”
			SO4: I always participate in extension activities and training conducted by farmer groups.	Likert scale, where 1 corresponded to “I strongly disagree” and 5 to “I strongly agree”

### Content validation

**Fig. 3:**
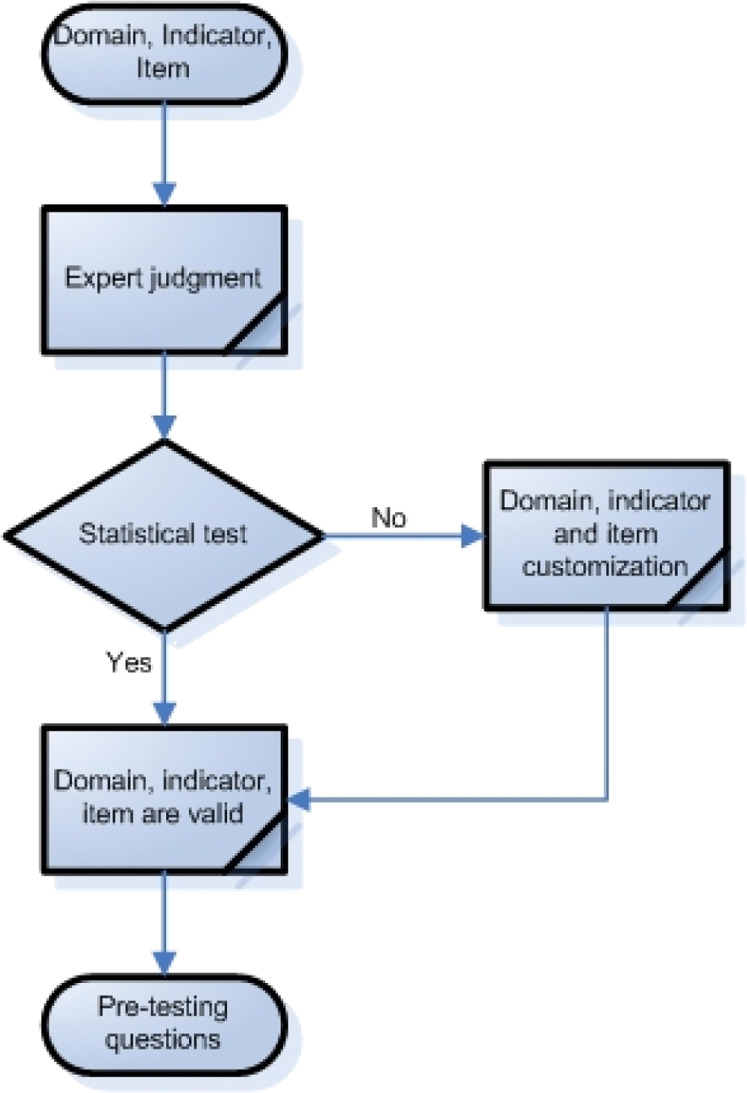
Content validation.

Content validation is required to ensure that only phenomena described in the conceptual definition are listed among the items and not other phenomena that are outside of the construct[26]. Content validation can be done using the expert judgment method. Expert judges are individuals with a high understanding of the domain and the scale being used[27]. Furthermore, they will evaluate each domain, indicator, and item used to measure the climate resilience of smallholder farmers (Fig. 3). This evaluation can be done using the Delphi statistical method[28].

### Pre-testing questions

**Fig. 4:**
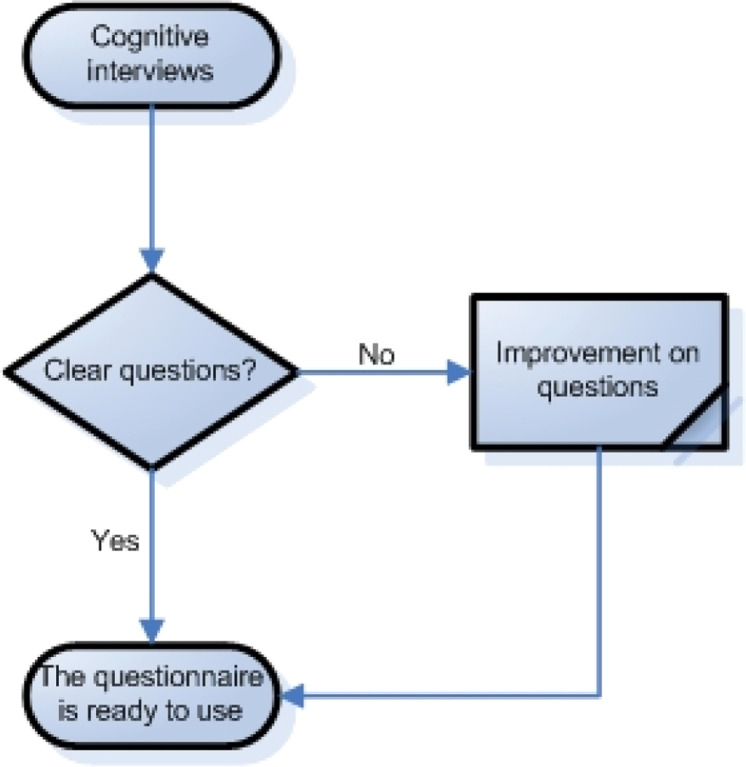
Pre-testing questions.

Then, domains, indicators, and items that have good content validity can be tested using pre-test questions. At this stage, the survey has not yet been implemented, meaning that the questions that have been compiled into a research questionnaire must be tested on potential respondents. The questionnaire can be tested by researchers using cognitive interviews. This stage allows a researcher to minimize the chances of misunderstanding and measurement errors in the actual survey[29]. Researchers can re-evaluate whether each item has used the correct sentence or expression to ensure the questions are clear and understandable (Fig. 4).

### Survey

**Fig. 5:**
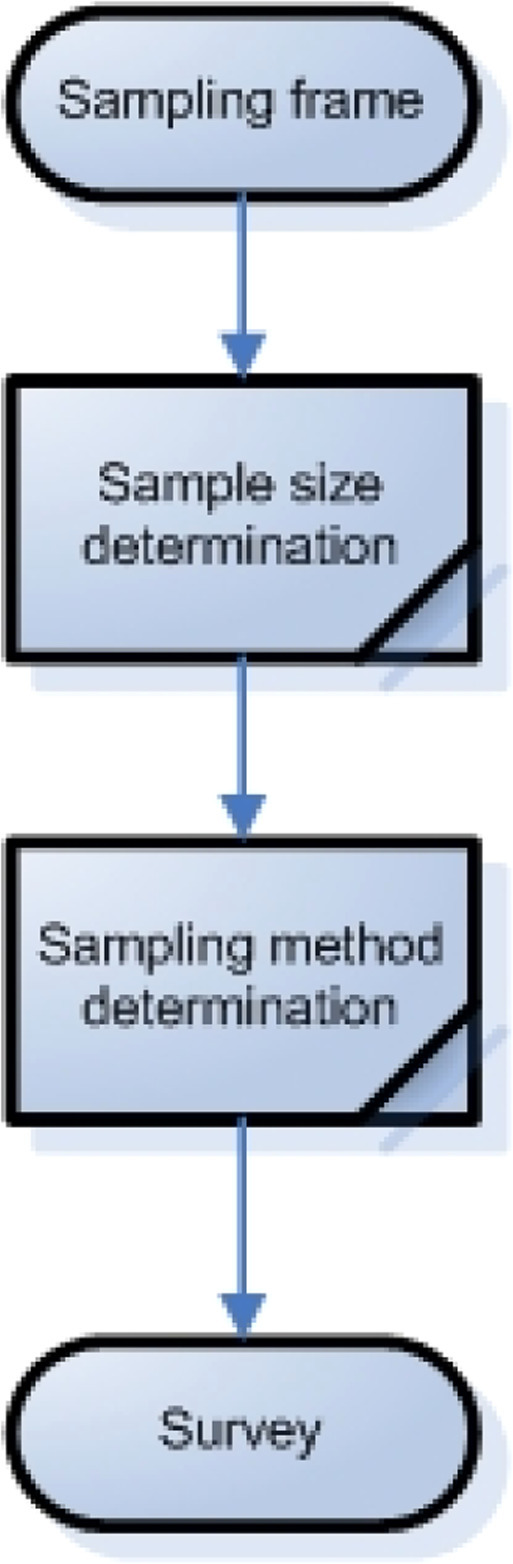
Steps in the survey stages.

At this stage, researchers need to prepare several things: survey equipment (e.g., questionnaires, stationery, etc.) and sampling frames (sampling size and methods) (Fig. 5). Survey equipment can be prepared by printing questionnaires or using a digital platform. In addition, the preparation of sampling frames can serve as a basis for determining sampling size. Determination of sampling size can be done using various methods, but the Slovin method is the most commonly used[30]. However, there is a general guideline in determining this sample size, such as using a sample of 10 respondents for each question item[31]. Some researchers also argue that 300 respondents is the best sample size[32], while others suggest an ideal sample of 200–300 respondents as a requirement for using factor analysis[33].

After that, researchers must decide on the sampling method to be used. Sampling methods can be divided into 2: probabilistic sampling and non-probabilistic sampling. The selection of both methods will depend on the availability of the sampling frame; if the sampling frame is available, it is advisable to use the probabilistic sampling method to reduce bias in data collection. However, if the sampling frame is not available properly, researchers can use the non-probabilistic sampling method. In this study, we selected 240 farmers who own and cultivate rice fields in Kebumen Regency, Indonesia, and were selected using the simple random sampling method.

### Item reduction analysis

**Fig. 6:**
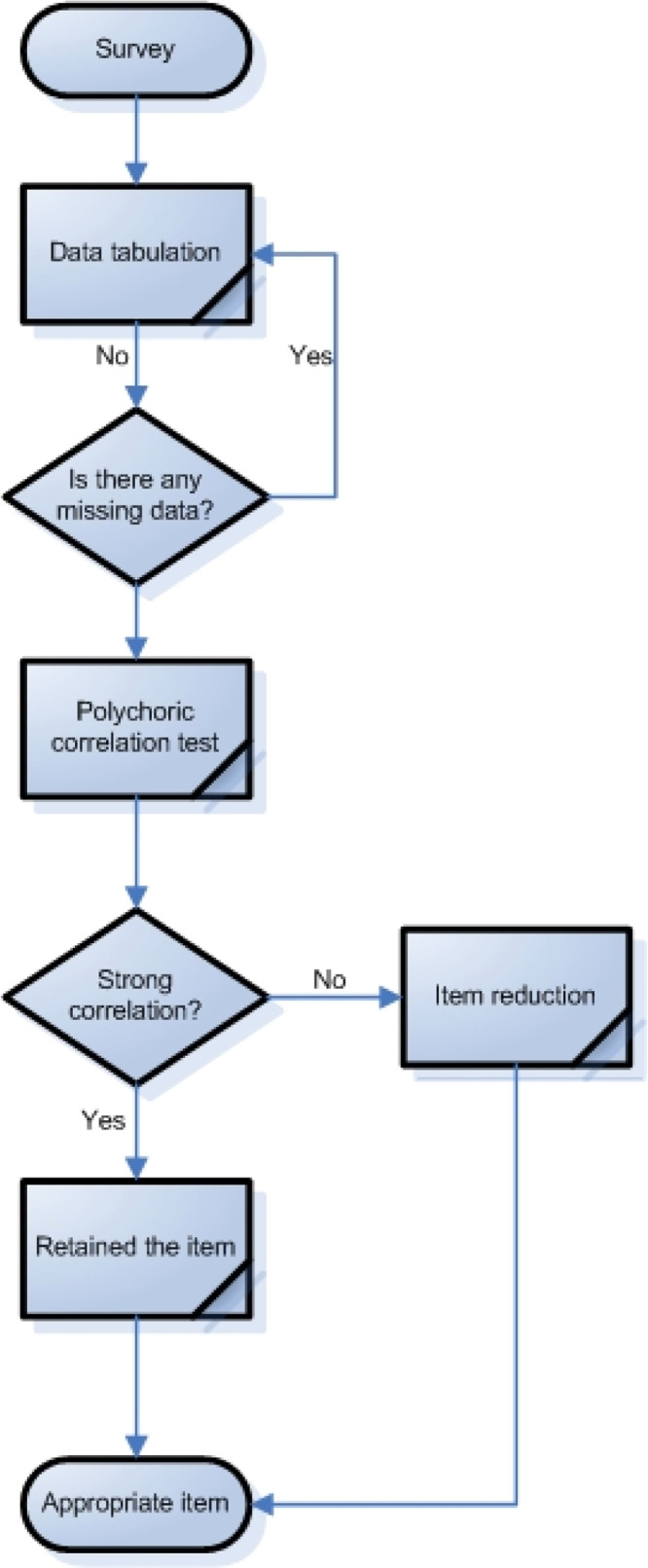
Item reduction analysis.

When the survey has been completed and the data have been collected, the next stage in assessing the smallholder farmers’ climate resilience is item reduction analysis. This stage must be carried out to re-ensure that only parsimonious, functional, and internally consistent items are used in the data analysis[34]. The first step that must be taken at this stage is to check for missing data. After that, a polychoric correlation test must be carried out to see whether there is one or more items that have a strong correlation (Fig. 6). Strong correlations between items in the same domain are preferred since they show that the item can measure the domain well[35]. To reduce items, it is possible to delete Items with a weak correlation (polychoric correlation coefficient < 0.30) as an alternative to be eliminated.

Survey data shows variations in each item that has been compiled (Tables 2, 3, 4). Since there are no missing data in the tabulated data, polychoric correlation analysis can be performed.

**Table 2:** Summary of data per item in the economic domain (EC).

Item	Score					Total
	Strongly Disagree	Disagree	Neutral	Agree	Strongly Agree	
EC1	18	136	16	42	28	240
EC2	13	151	18	48	10	240
EC3	18	151	22	36	13	240
EC4	19	166	21	23	11	240
EC5	17	153	20	41	9	240

Source: Primary data analysis, 2025.

**Table 3:** Summary of data per item in the environment domain (EN).

Item	Score					Total
	Strongly Disagree	Disagree	Neutral	Agree	Strongly Agree	
EN1	19	143	19	56	3	240
EN2	24	147	19	43	7	240
EN3	18	161	15	28	18	240
EN4	3	29	8	166	34	240
EN5	8	37	13	135	47	240
EN6	19	166	21	23	11	240

Source: Primary data analysis, 2025.

**Table 4:** Summary of data per item on the social domain (SO).

Item	Score					Total
	Strongly Disagree	Disagree	Neutral	Agree	Strongly Agree	
SO1	5	65	6	151	13	240
SO2	7	22	8	185	18	240
SO3	3	63	13	148	13	240
SO4	10	36	9	168	17	240

Source: Primary data analysis, 2025.

According to the polychoric test, there are items in the economic domain (EC) that have a strong correlation with each other (Table 5). However, several items in the environmental domain (EN) appear to have a weak correlation (polychoric correlation value on items < 0.30) (Table 6), so these items need to be eliminated (EN1, EN4, and EN5) to increase the robustness of the constructed domain (Table 7). Furthermore, the social domain (SO) has a high polychoric correlation score (Table 8).

**Table 5:** Polychoric correlation test for the economic domain (EC).

Polychoric correlation	EC1	EC2	EC3	EC4	EC5
EC1	1.00				
EC2	0.57	1.00			
EC3	0.74	0.68	1.00		
EC4	0.84	0.78	0.90	1.00	
EC5	0.72	0.73	0.78	0.89	1.00

Source: Primary data analysis, 2025.

**Table 6:** Polychoric correlation test for the environment domain (EN) before item reduction.

Polychoric correlation	EN1	EN2	EN3	EN4	EN5	EN6
EN1	1.00					
EN2	0.77	1.00				
EN3	0.55	0.64	1.00			
EN4	0.12	0.11	0.08	1.00		
EN5	0.07	0.08	0.04	0.77	1.00	
EN6	0.71	0.85	0.79	0.07	0.02	1.00

Source: Primary data analysis, 2025.

**Table 7:** Polychoric correlation test for the environment domain (EN) after item reduction.

Polychoric correlation	EN2	EN3	EN6
EN2	1.00		
EN3	0.64	1.00	
EN6	0.85	0.79	1.00

Source: Primary data analysis, 2025.

**Table 8:** Polychoric correlation test for the social domain (SO).

Polychoric correlation	SO1	SO2	SO3	SO4
SO1	1.00			
SO2	0.75	1.00		
SO3	0.53	0.76	1.00	
SO4	0.57	0.84	0.60	1.00

Source: Primary data analysis, 2025.

### Extraction of factors

Factor extraction is one of the important stages in constructing a multidimensional domain. At this stage, a researcher can determine the optimal number of factors, or what are known as domains, that can be used in the assessment[36]. Optimal domains can be done using factor analysis. Factors must be maintained or said to be optimal when they have an Eigenvalue greater than 1[37] (Table 9.). Based on the climate resilience assessment findings, three domains must be maintained. Therefore, the economic (EC), environment (EN), and social (SO) domains can be used well to measure the smallholder farmers’ climate resilience.

**Table 9:** Factor analysis results.

Factor	Eigenvalue	Difference	Proportion	Cumulative
Factor1	6.13	2.96	0.50	0.50
Factor2	3.17	1.16	0.26	0.77
Factor3	2.00	1.48	0.16	0.93
Factor4	0.53	0.05	0.04	0.97
Factor5	0.48	0.15	0.04	1.01
LR test				53.21
Prob>chi2				0.00

Source: Primary data analysis, 2025.

### Tests of dimensionality

**Fig. 7:**
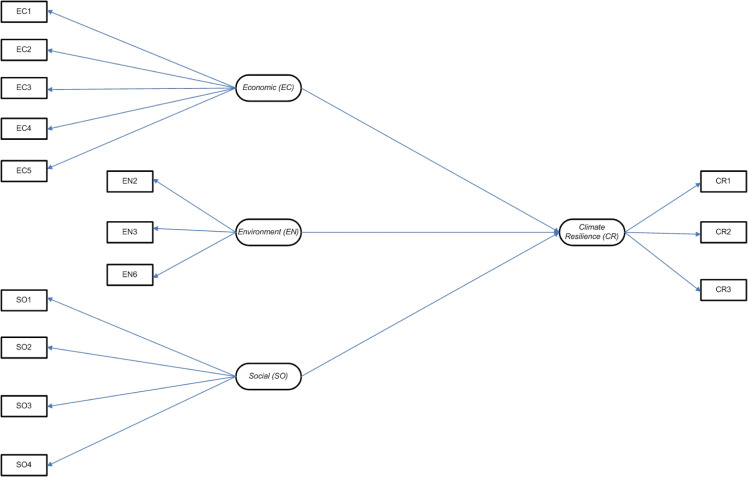
Construction of a climate resilience model of smallholder farmers.

The dimensionality tests are conducted to find out how much each constructed domain contributes to the overall model construction. At this stage, each domain that has been formed must be further analyzed using factor loading analysis. A domain can be said to have a good contribution to the constructed model when it has a factor loading value of >0.70[38]. The dimensionality test findings (Fig. 7) show that all domains can provide a good contribution (factor loading value > 0.70) (Table 10).

**Table 10:** Factors loading analysis.

Item/Domain	Economic (EC)	Environment (EN)	Social (SO)
EC1	0.79		
EC2	0.77		
EC3	0.89		
EC4	0.96		
EC5	0.87		
EN2		0.88	
EN3		0.82	
EN6		0.92	
SO1			0.73
SO2			0.95
SO3			0.71
SO4			0.87

Source: Primary data analysis, 2025.

## Protocol validation

A robust multidimensional domain has been formed if all stages in this protocol have been carried out correctly. Furthermore, to validate the protocol, it can be done by examining the reliability and validity of the construct model of smallholder farmers’ climate resilience.

### Tests of reliability

Reliability can be interpreted as the degree of consistency of a measurement tool when a measurement is repeated under the same conditions. In this study, reliability refers to the degree of consistency of the scale used to measure the smallholder farmers’ climate resilience to each item in each domain (Table 11). Reliability tests can be done using the Cronbach's alpha method, where the alpha coefficient of 0.70 is considered to be the ideal threshold for demonstrating the reliability of each scale used in each domain[15].

**Table 11:** Tests of reliability.

Domain	Cronbach's Alpha
Economic (EC)	0.911
Environment (EN)	0.847
Social (SO)	0.838

Source: Primary data analysis, 2025.

### Tests of validity

Domain validity testing can be done using various methods. The discriminant validity is one of the simplest and most popular methods. By using this technique, a researcher can ascertain whether a developed construct has a certain uniqueness that is different from other constructs. In other words, the constructed construct does not measure the same thing as other constructs[39]. The Fornell-Larcker criterion was used to examine domain validity in the smallholder farmers’ climate resilience construct model. The construct in this study is declared valid because the diagonal column value is higher than the value in each domain column[40] (Table 12.).

**Table 12:** Test of validity.

Domain	Economic (EC)	Environment (EN)	Social (SO)
Economic (EC)	0.86		
Environment (EN)	0.52	0.88	
Social (SO)	0.05	0.06	0.82

Source: Primary data analysis, 2025.

## Limitations

This study focuses on agricultural activities carried out by farmers who own and cultivate land simultaneously. Furthermore, this model was initiated with the target of farmers involved in rice farming.

## CRediT author statement

All Authors were involved in preparing the manuscript.

## CRediT authorship contribution statement

**Imade Yoga Prasada:** Conceptualization, Methodology, Data curation, Formal analysis, Writing – original draft, Writing – review & editing. **Jangkung Handoyo Mulyo:** Conceptualization, Formal analysis, Methodology, Writing – original draft. **Hani Perwitasari:** Visualization, Supervision, Writing – original draft. **Agus Dwi Nugroho:** Methodology, Validation, Writing – review & editing.

## Declaration of competing interest

The authors declare that they have no known competing financial interests or personal relationships that could have appeared to influence the work reported in this paper.

## Data Availability

Data will be made available on request.

## References

[1] Folke C., Carpenter S.R., Walker B., Scheffer M., Chapin T., Rockström J. (2010). Resilience thinking: integrating Resilience, adaptability and transformability. Ecol. Soc..

[2] Mahajan S., Hausladen C.I., Argota Sánchez-Vaquerizo J., Korecki M., Helbing D. (2022). Participatory resilience: surviving, recovering and improving together. Sustain. Cities Soc..

[3] Kourtit K., Nijkamp P., Banica A. (2023). An analysis of natural disasters’ effects – A global comparative study of ‘Blessing in Disguise. Socioecon. Plann. Sci. [Internet].

[4] Holling C.S. (1973). Resilience and stability of ecological systems. Annu. Rev. Ecol. Syst..

[5] Berkes F., Folke C. (1998). https://www.cambridge.org/vi/universitypress/subjects/life-sciences/ecology-and-conservation/linking-social-and-ecological-systems-management-practices-and-social-mechanisms-building-resilience?format=PB.

[6] Majale M. Towards Pro-Poor Regulatory Guidelines for Urban Upgrading. Bourton Hall, UK; 2002.

[7] Giesselmann J., Sikstel A. (2025). A-posteriori error estimates for systems of hyperbolic conservation laws via computing negative norms of local residuals. IMA J. Numer. Anal..

[8] Mulargia F. (2001). Retrospective selection bias (or the benefit of hindsight). Geophys. J. Int..

[9] Tyrer F., Bhaskaran K., Rutherford M.J. (2022). Immortal time bias for life-long conditions in retrospective observational studies using electronic health records. BMC Med. Res. Methodol. [Internet].

[10] Beltz A.M., Molenaar P.C. (2015). A posteriori model validation for the temporal order of directed functional connectivity maps. Front. Neurosci..

[11] Smith E., Xiao Y., Xie H., Manwaring S.S., Farmer C., Thompson L. (2023). Posterior superior temporal cortex connectivity is related to social communication in toddlers. Infant. Behav. Dev..

[12] Cignarale G., Schmid U., Tahko T., Kuznets R. (2023). The role of A Priori belief in the design and analysis of fault-tolerant distributed systems. Minds Mach. [Internet]..

[13] Weber-Stadlbauer U., Meyer U. (2019). Challenges and opportunities of a-priori and a-posteriori variability in maternal immune activation models. Curr. Opin. Behav. Sci..

[14] Palafoutas J., Velasco Romero D.A., Teyssier R. (2025). Comparison between a priori and a posteriori slope limiters for high-order finite volume schemes. J. Comput. Phys..

[15] Boateng G.O., Neilands T.B., Frongillo E.A., HR Melgar-Quiñonez, SL Young (2018). Best practices for developing and validating scales for health, social, and behavioral research: a primer. Front. Public Heal [Internet].

[16] Kock F., Berbekova A., Assaf A.G. (2021). Understanding and managing the threat of common method bias: detection, prevention and control. Tour. Manag. [Internet]..

[17] Valery Chirkov, Jade Anderson (2018). Statistical positivism versus critical scientific realism. A comparison of two paradigms for motivation research: part 1. A philosophical and empirical analysis of statistical positivism. Theory Psychol..

[18] Wiwoho G., Prasada I.Y. (2024). Geomembrane-based salt production method to increase the quantity and quality of small-scale salt producer. MethodsX [Internet].

[19] Kumar Shrotryia Vijay, Upasana Dhanda (2019). Content validity of assessment instrument for employee engagement. SAGE Open [Internet].

[20] Atkins L., Francis J., Islam R., O’Connor D., Patey A., Ivers N. (2017). A guide to using the Theoretical Domains Framework of behaviour change to investigate implementation problems. Implement. Sci..

[21] Carstensen C., Khot R., Pani A.K. (2022). A priori and a posteriori error analysis of the lowest-order NCVEM for second-order linear indefinite elliptic problems. Numer. Math..

[22] Hinkin T.R. (1995). A review of scale development practices in the study of organizations. J. Manage..

[23] Hair JF L.D.S., Gabriel M., da Silva D., Braga Junior S. (2019). Development and validation of attitudes measurement scales: fundamental and practical aspects. RAUSP Manag. J..

[24] Dixon J.L., Stringer L.C., Challinor A.J. (2014). Farming system evolution and adaptive capacity: insights for adaptation support. Resources.

[25] Purvis B., Mao Y., Robinson D. (2019). Three pillars of sustainability: in search of conceptual origins. Sustain. Sci..

[26] DeVellis R.F. (2012).

[27] Morgado F.F.R., Meireles J.F.F., Neves C.M., Amaral A.C.S., Ferreira M.E.C. (2017). Scale development: ten main limitations and recommendations to improve future research practices. Psicol Reflexão e Crítica.

[28] Taghipoorreyneh M., Tierney R.J., Rizvi F. (2023). Ercikan KBTIE of E (Fourth E.

[29] Hannan A., Heckert J., James-Hawkins L., Yount K.M (2020). Cognitive interviewing to improve women’s empowerment questions in surveys: application to the health and nutrition and intrahousehold relationships modules for the project-level Women’s Empowerment in Agriculture Index. Matern. Child. Nutr..

[30] Ayembilla J.A., Quarcoo A., Whyte B.K., Gordon A., Otu P.N.Y., Bonah D.A. (2024). Health risk assessment of potassium bromate in bread in Ghana. Heliyon [Internet].

[31] Nunnally J.C (1978).

[32] Clark L.A., Watson D. (1995).

[33] Guadagnoli E., Velicer W.F. (1988).

[34] Thurstone L.L. (1947).

[35] Piedmont R.L., Michalos A.C. (2014). Inter-item Correlations BT - Encyclopedia of Quality of Life and Well-Being Research.

[36] Maskey R., Fei J., Nguyen H.O (2018). Use of exploratory factor analysis in maritime research. Asian J. Shipp. Logist. [Internet].

[37] Patil V.H., Singh S.N., Mishra S., Todd Donavan D. (2008). Efficient theory development and factor retention criteria: abandon the ‘eigenvalue greater than one’ criterion. J. Bus. Res..

[38] Liao H.C., Huang C.Y., Wang Y.H. (2022). Development and psychometric testing of a scale measuring caring behaviors for healthcare students and providers. Med. Educ. Online.

[39] Clark L.A., Watson D. (2019). Constructing validity: new developments in creating objective measuring instruments. Psychol. Assess..

[40] Mushi H.M. (2024). Digital marketing strategies and SMEs performance in Tanzania: insights, impact, and implications. Cogent. Bus. Manag. [Internet].

